# LncRNA LUCAT1 facilitates tumorigenesis and metastasis of triple-negative breast cancer through modulating miR-5702

**DOI:** 10.1042/BSR20190489

**Published:** 2019-09-03

**Authors:** Exian Mou, Hao Wang

**Affiliations:** Breast Surgery, Sichuan Cancer Hospital & Institute, Sichuan Cancer Center, School of Medicine, University of Electronic Science and Technology of China, Chengdu, Sichuan 610041, China

**Keywords:** lncRNA, LUCAT1, metastasis, miR-5702, triple-negative breast cancer, tumorigenesis

## Abstract

Triple-negative breast cancer (TNBC) is a subtype of aggressive breast cancer with high recurrence and poor survival. Emerging evidence has indicated that long non-coding RNAs (lncRNAs) play pivotal roles in the development and progression of multiple cancers. Although there are substantial studies revealing that lung cancer-associated transcript 1 (LUCAT1) functions as a tumor promotor in various human cancers, the molecular mechanism of LUCAT1 in TNBC remains largely to be explored. In our study, we identified that LUCAT1 expression was dramatically enhanced in TNBC samples and cells. High LUCAT1 expression was strongly associated with advanced stages and poor prognosis of TNBC. LUCAT1 contributed to TNBC development through accelerating cell proliferation, cell cycle progression and metastasis as well as attenuating cell apoptosis. Moreover, miR-5702 was proved to directly bind to LUCAT1 and be negatively modulated by LUCAT1. Knockdown of miR-5702 reversed the suppressing influences of LUCAT1 depletion on TNBC progression. In conclusion, it was the first investigation to shed light on the significant function and underlying regulatory mechanism of LUCAT1 in TNBC tumorigenesis. We validated that LUCAT1 induced tumorigenesis and metastasis of TNBC via miR-5702, which provided clues for improving the treatment of TNBC.

## Introduction

Breast cancer remains the most prevalent diagnosed malignant neoplasm and ranks the first leading cause of cancer-related death among women worldwide [1,2]. It is well known that breast cancer is a heterogeneous disease whose gene-expression profiles are various between individuals [3]. Triple-negative breast cancer (TNBC) is a kind of breast cancer, which accounts for approximately 20% of whole breast cancers, and more aggressive than other subtypes of breast cancer because of its higher recurrence and poorer outcomes [4,5]. TNBC is characterized by absence of estrogen receptor (ER), progesterone receptor (PR) along with human epidermal growth factor receptor-2 (HER2) [6]. It has been reported ER, PR, and HER2 are of immense importance in the clinical diagnosis and therapy for breast cancer [7]. For lacking ER, PR, and HER2 expression, there are only a few effective adjuvant treatments of patients with TNBC, including conventional surgery, radiotherapy, and chemotherapy [8]. Accordingly, it is of great clinical significance to develop reliable molecular therapeutic targets for TNBC patients.

In recent years, long non-coding RNAs (lncRNAs) have gained mounting attention in cancer investigations [9]. LncRNAs are defined as a category of non-protein coding transcripts which are generally greater than 200 nucleotides in length [10]. A growing body of studies has demonstrated that lncRNA plays a pivotal role in the development and progression of multiple cancers. For instance, lncRNA SLCO4A1-AS1 facilitates growth and metastasis of colorectal cancer through β-catenin-dependent Wnt pathway [11]. LncRNA TUG1 promotes cells proliferation and inhibits cells apoptosis through regulating AURKA in epithelial ovarian cancer cells [12]. LncRNA PVT1 promotes ovarian cancer progression by silencing miR-214 [13]. Previous researches have substantial evidence that lncRNA lung cancer-associated transcript 1 (LUCAT1) is recognized as a tumor promoting gene in a wide range of human cancers. For example, LUCAT1 is associated with poor prognosis in human non-small lung cancer and regulates cell proliferation via epigenetically repressing p21 and p57 expression [14]. Knockdown of long non-coding RNA LUCAT1 inhibits cell viability and invasion by regulating miR-375 in glioma [15]. LUCAT1 promotes malignancy of ovarian cancer through regulation of miR-612/HOXA13 pathway [16]. Nevertheless, the biological function and underlying mechanism of LUCAT1 in TNBC have yet been elucidated.

It is evident from extensive investigations that lncRNAs can serve as ceRNAs similar to miRNA sponges and thereby participate in cancer development [17]. Long non-coding RNA MIR31HG inhibits hepatocellular carcinoma proliferation and metastasis by sponging microRNA-575 to modulate ST7L expression [18]. LncRNA NEAT1 accelerates lung adenocarcinoma deterioration and binds to mir-193a-3p as a competitive endogenous RNA [19]. In the present study, we found that there were potential binding sites between miR-5702 and LUCAT1 by lncBase v.2 database in DIANA tools. As a result, we probed the regulatory mechanism of LUCAT1 in TNBC through investigating LUCAT1/miR-5702 axis.

Our study delineated that LUCAT1 expression was increased and closely associated with poor prognosis in TNBC. Further, LUCAT1 promoted tumorigenesis and metastasis of TNBC via miR-5702. Hence, the LUCAT1/miR-5702 axis appeared to be a novel promising therapeutic target for TNBC patients.

## Materials and methods

### Clinical sample collection

Ninety-four pairs of TNBC samples and matched adjacent normal samples were obtained from TNBC patients who underwent surgery at Sichuan Cancer Hospital & Institute, Sichuan Cancer Center, School of Medicine, University of Electronic Science and Technology of China. All participants were women and their ages range from 40 to 65. The mean age of all patients was 52.213. There were 72 patients at tumor stage I/II, including 35 patients with low LUCAT1 expression and 37 patients with high LUCAT1 expression. Furthermore, 22 patients were at tumor stage III/IV, among which there were 12 patients with low LUCAT1 level and 10 patients with high LUCAT1 level. None of enrolled patients received any cancer treatment prior to surgical resection. All clinical samples were conserved at −80°C until consequent use. The present study was conducted with permission by Ethics Review Committees of Sichuan Cancer Hospital & Institute, Sichuan Cancer Center, School of Medicine, University of Electronic Science and Technology of China. All tissues were collected with written inform consent from patients and confirmed on the grounds of postoperative histopathological examination and immunohistochemistry.

### Cell culture

Human immortalized breast epithelial cell line MCF-10A and TNBC cell lines (MDA-MB-231, TB-549, MDA-MB-453, MDA-MB-468) were purchased from the American Type Culture Collection (ATCC, Manassas, VA, U.S.A.). All TNBC cell lines were maintained in RPMI-1640 medium (Gibco, Carlsbad, CA, U.S.A.) containing 10% fetal bovine serum (FBS, Gibco), 100 U/ml penicillin (Gibco), and 100 U/ml streptomycin (Gibco). MCF10A cell line was grown in DMEM/F12 (Invitrogen, Carlsbad, CA, U.S.A.) with 5% horse serum, 20 ng/ml epidermal growth factor, 0.5 μg/ml hydrocortisone, 10 μg/ml insulin, 100 μg/ml penicillin–streptomycin, and 100 ng/ml cholera toxin. All cells were cultured at 37°C in a humidified condition supplemented with 5% CO_2_.

### Cell transfection

The short hairpin RNA targeting LUCAT1 (shLUCAT1) and a scrambled shRNA as negative control (shCtrl) were obtained from Invitrogen (Invitrogen, MA, U.S.A.). In addition, miR-5702 mimics, miR-5702 inhibitors and matched negative control (miR-NC) were synthesized by Ribobio (Guangzhou, China). Cells were transfected with corresponding plasmids by using Lipofectamine 2000 (Invitrogen) following the product instructions. After transfection for 48 h, cells were harvested for further experiments. The involved sequences were as follows: shLUCAT1 (sense): 5′-CCCAUCAGAAGAUGUCAGAAGAUAA-3′, (antisense): 5′-UUAU CUUCUGA CAUCUUCUGAUGGG-3′.

### Quantitative real-time PCR

Total RNA was isolated from clinical tissues or cultured cells by the Trizol reagent (Invitrogen, Carlsbad, U.S.A.). In line with the manufacturer’s instructions, reverse transcription was performed with the Prime Script™ RT reagent kit (Takara Biotechnology, China). Subsequently, quantitative real-time PCR (qRT-PCR) was utilized to quantify target genes with SYBR Premix Ex Taq II (Takara Biotechnology, China) on a CFX96 Real-Time PCR Detection System (Bio-Rad, CA, U.S.A.). GAPDH and U6 were respectively employed as internal controls. Relative expression levels of genes were determined by using the 2^‒ΔΔ*C*_t_^ method. The primers used for qRT-PCR were presented as follows:

LUCAT1: 5′-ACCAGCTGTCCCTCAGTGTTCT-3′ (sense),

5′-AGGCCTTTATCCTCGGGTTGCCT-3′ (antisense);

miR-5702: 5′-UGAGUCAGCAACAUUAU-3′ (sense),

5′-GTGCAGGGT CCGAGGT-3′ (antisense);

U6: 5′-CTCGCTTCGGCAGCACA-3′ (sense),

5′-AACGCTTCACGAATTTGCGT-3′ (antisense);

GAPDH: 5′-GTCAA CGG ATTTGGTCTGTATT-3′ (sense),

5′-AGTCTTCTGGGTGGCAGTAT-3′ (antisense).

### Cell counting kit‐8 assay

Cell proliferation was estimated by the Cell Counting Kit-8 (CCK-8; Sigma, Santa Clara, CA, U.S.A.) assay. Transfected cells were plated into 96‐well plates at a density of 2000 cells per well. After incubation for 0, 24, 48, 72 h, 10 μl CCK‐8 solution (Dojindo, Japan) was added to each well and then cells were cultured at 37°C for 4 h. Cellular viability was detected by absorbance at 450 nm utilizing a microplate reader (Bio-Rad, Hercules, CA, U.S.A.).

### Ethynyldeoxyuridine assay

The ethynyldeoxyuridine (EdU) assay was conducted with the EdU labeling/detection kit (Ribobio, Guangzhou, China) based on the manufacturer’s protocol. After transfection, cells were seeded at the concentration of 5 × 10^3^ per well into 6-well plates and then incubated with 50 μM EdU labeling medium for 2 h at 37°C. Then, cells were fixed in 4% paraformaldehyde, treated with 0.5% Triton X-100, stained by anti-EdU working solution, and followed by incubation with 100 μl DAPI. The percentage of EdU-positive cells was counted in five random fields of view using fluorescent microscopy. This assay was repeated three times.

### Flow cytometry assay

For cell cycle analysis, cells were collected by trypsinization and fixed in 70% ethanol. After eluting with PBS, cells were resuspended in 0.2 ml of PI staining solution with 50 μg/ml PI and 100 μg/ml RNase A, followed by incubation at room temperature for 1 h and then analyzed by a BD LSRFortessaTM flow cytometer (BD Biosciences, Bedford, MA, U.S.A.).

For cell apoptosis analysis, APC Annexin V Apoptosis Detection Kit (BioLegend, San Diego, CA, U.S.A.) was used to identify apoptotic cells. Transfected cells were harvested and rinsed twice by PBS. Collected cells were double stained using fluorescein isothiocyanate-Annexin V and propidium iodide. Then, cell apoptotic rate was analyzed by using a flow cytometer equipped with CellQuest software (BD Biosciences).

### Transwell assays

Transwell assays were carried out by using transwell chamber inserts (8 μm pore size; Corning Inc., Corning, NY, U.S.A.) and matrigel (24 well inserts; BD, Franklin Lakes, U.S.A.) to measure the properties of cell migration and invasion. For cell migration, Cells in serum-free medium were added to the upper chamber of transwell inserts pre-coated with matrigel for cell invasion assay or without matrigel for cell migration assay. The bottom chamber was supplemented with complete medium. After 24 h of incubation, cells in upper chambers were discarded, and migrated or invaded cells were fixed in methanol and stained with 0.1% crystal violet. The cell numbers were counted under a microscope in five different random fields.

### Western blotting assay

Cells were dissolved in RIPA buffer (Thermo Fisher Scientific, Waltham, MA, U.S.A.) with the protease inhibitor cocktail (Roche, CA, U.S.A.). Total protein concentration was measured by the Bio-Rad assay system (Bio-Rad Laboratories, Hercules, CA, U.S.A.). Total protein extract (40 μg) was separated by electrophoresis on 10% SDS gel and then transferred to PVDF (Merck Millipore, Burlington, MA, U.S.A.). PVDF membranes were sealed in 5% skim milk and subsequently incubated with primary antibodies at 4°C overnight. After rinsing with TBST three times, membranes were incubated with secondary antibody at room temperature for 2 h. The blots were determined by an enhanced chemiluminescence kit (Merck Millipore) using a Molecular Imager system (Bio-Rad Laboratories). GAPDH acted as the internal reference. The specific primary antibodies for E-cadherin (ab40772), N-cadherin (ab76011), and GAPDH (ab9485) were purchased from Abcam.

### Luciferase reporter assays

The wide-type and mutant sequences of LUCAT1 were cloned into pmirGLO Dual-luciferase vectors (Promega, Madison, WI, U.S.A.) to construct the reporter vectors LUCAT1-WT and LUCAT1-Mut. The resulting constructs were co-transfected with miR-5702 mimics or negative control (miR-NC) into TNBC cells using Lipofectamine 2000. After 48 h of transfection, relative luciferase activities were determined by a dual-luciferase reporter assay system (Promega) on the basis of the manufacturer’s guide.

### RNA immunoprecipitation assay

In accordance with product manuals, RNA immunoprecipitation (RIP) assays were performed with EZMagna RIP RNA-binding protein immunoprecipitation kit (Millipore, U.S.A.). Cells lysate was maintained in RIP buffer with magnetic beads conjugated with Ago2 antibodies (Millipore, U.S.A.). IgG served as the negative control. After 2 h of incubation at 4°C, coprecipitated RNAs were subjected to PCR analysis.

### RNA pull-down assay

The Pierce RNA 3′ End Desthiobiotinylation Kit (Thermo Fisher Scientific) was applied to generate biotinylated miRNAs. Briefly, cell lysates were incubated with biotin-labeled miR-NC, miR-5702-WT, or miR-5702-Mut and magnetic beads for 2 h at 37°C. Afterwards, the beads were harvested and washed. Then, precipitated RNAs were eluted, purified, and detected using qRT-PCR assay.

### Statistical analysis

Statistical analyses were conducted using SPSS 20.0 software (SPSS Inc., U.S.A.) and all data were displayed as mean ± standard deviation. Student’s *t*-test and one-way ANOVA were applied to evaluate the differences between groups. Overall survival curves were expressed by the Kaplan–Meier analysis using the log-rank test. Correlations between study objects were assessed by Spearman’s rank correlation coefficients. *P*<0.05 was defined as statistically significant. Each assay was repeated at least three times.

## Results

### LUCAT1 expression is notably enhanced and closely associated with poor prognosis in TNBC

**Figure 1:**
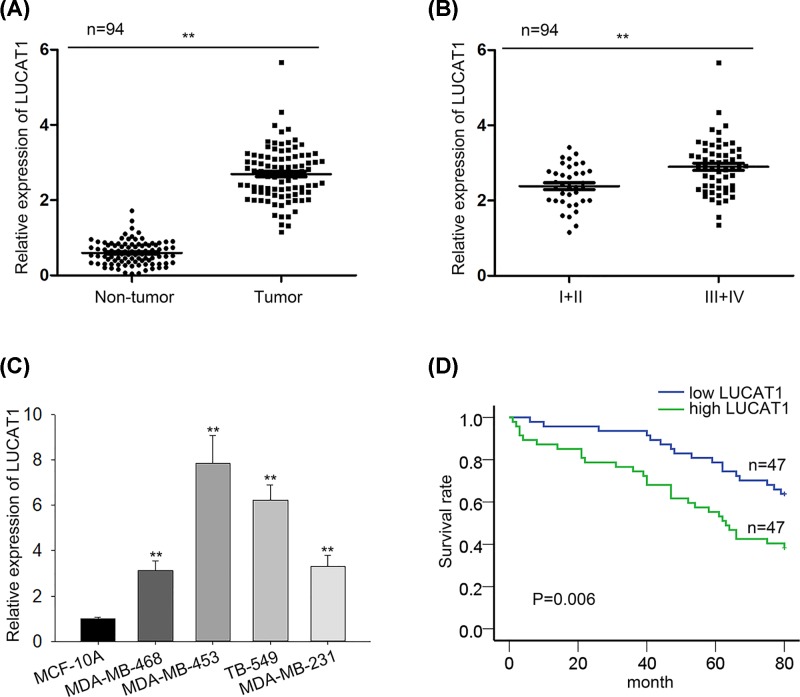
LUCAT1 expression is notably enhanced and closely associated with poor prognosis in TNBC (**A**–**C**) The qRT-PCR analysis was applied to detect the expression of LUCAT1 in clinical tissues and cell lines as well as in different TNBC stages. (**D**) Overall survival curve was plotted by Kaplan–Meier analysis on basis of LUCAT1 expression in patients with TNBC. All data were expressed as the mean ± SD, **P<0.01.

Mounting evidence has corroborated that LUCAT1 exerts its oncogenic function in a variety of cancers. In order to probe the biological role of LUCAT1 in TNBC progression, we measured the expression of LUCAT1 in 94 recruited TNBC specimens and corresponding adjacent specimens. Our investigation exhibited that LUCAT1 expression was prominently increased in TNBC tissues compared to matched non-tumor tissues (Figure 1A). Furthermore, the obvious rise of LUCAT1 expression was observed in advanced clinical stage patients with TNBC (Figure 1B). The analysis of clinical data manifested that LUCAT1 expression was correlated with tumor grade, lymph node metastasis, and distant metastasis (Table 1). In concert with these results, the expression level of LUCAT1 in TNBC cell lines (MDA-MB-231, TB-549, MDA-MB-453, MDA-MB-468) was higher than that in immortalized breast epithelial cell line MCF-10A (Figure 1C). Meanwhile, our data from Kaplan–Meier survival analysis revealed TNBC patients with high LUCAT1 expression level presented much worse overall survival than those with low expression of LUCAT1 (Figure 1D). Multivariate analysis suggested that tumor grade and LUCAT1 expression level were independent prognostic factors of TNBC (Table 2). To sum up, LUCAT1 expression level is markedly increased and strongly correlated with poor prognosis.

**Table 1:** Association between clinical features and LUCAT1 expression of TNBC patients (*n*=94)

Variable	LUCAT1 expression		*P*-value
	Low	High	
**Age**			
<55	35	37	0.808
≥55	12	10	
**Menopause**			
No	37	34	0.631
Yes	10	13	
**Tumor size**			
<3	30	28	0.832
≥3	17	19	
**Tumor grade**			
I	24	13	0.034*
II/III	23	34	
**Stage**			
I/II	35	37	0.808
III/IV	12	10	
**Lymph node metastasis**			
No	29	16	0.013
Yes	18	31	
**Distant metastasis**			
No	30	14	0.002**
Yes	17	33	

Low or high was evaluated by the sample mean of LUCAT1 expression. Pearson χ^2^ test was employed to analyze clinical data. *P<0.05, **P<0.01.

**Table 2:** Multivariate analysis of prognostic parameters in TNBC patients by Cox regression analysis

Variable	Category	*P*-value
**Age**	<55	0.251
	≥55	
**Menopause**	No	0.351
	Yes	
**Tumor size**	<3	0.101
	≥3	
**Tumor grade**	I	0.023*
	II/III	
**Stage**	I/II	0.424
	III/IV	
**Lymph node metastasis**	No	0.739
	Yes	
**Distant metastasis**	No	0.884
	Yes	
**LUCAT1 level**	Low	0.025*
	High	

Proportional hazards method analysis revealed the importance of tumor grade and LUCAT1 level in independent prognostic factors of TNBC. *P<0.05.

### Knockdown of LUCAT1 suppresses cell proliferation and facilitates cell apoptosis in TNBC

**Figure 2:**
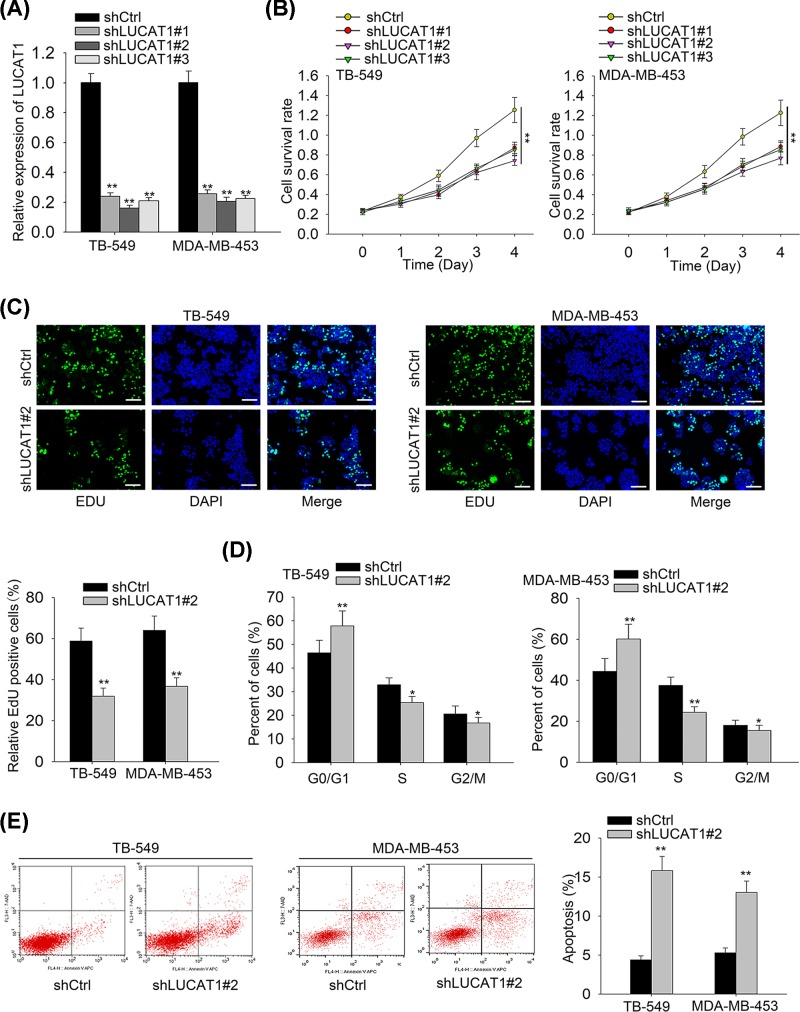
Knockdown of LUCAT1 suppresses cell proliferation and facilitates cell apoptosis in TNBC (**A**) The expression of LUCAT1 in TNBC cells was down-regulated by using shLUCAT1 and assessed by qRT-PCR. (**B,C**) Cell proliferation was estimated by CCK-8 and EdU assays. Scale bar = 100 μm. (**D,E**) Flow cytometry assay was carried out to analyze cell cycle and apoptosis rate. All data were expressed as the mean ± SD, *P<0.05, **P<0.01.

To estimate the influences of LUCAT1 on TNBC progression, we first down-regulated the expression of LUCAT1 by transfection with designed shLUCAT1 and then conducted the qRT-PCR analysis to determine the transfection efficiency. The results from qRT-PCR implied that LUCAT1 expression was significantly reduced in TNBC cell lines after transfection with shLUCAT1#1/2/3 vectors (Figure 2A). The CCK-8 and EdU assays were applied to evaluate cell proliferative capacity and our data displayed that silenced LUCAT1 notably repressed cell proliferation in TNBC (Figure 2B,C). The cell cycle assay analyzed by flow cytometry demonstrated that knockdown of LUCAT1 remarkably enhanced the percentage of cells in G0/G1 phase and declined the cell percentage in S phase (Figure 2D). Concordant with results above, we discovered that LUCAT1 silence elevated cell apoptosis of TNBC as compared with negative control (Figure 2E). Collectively, LUCAT1 knockdown inhibits cell proliferation and cell cycle progression whereas promotes cell apoptosis of TNBC.

### LUCAT1 suppression inhibits cell migration, invasion, and EMT of TNBC

**Figure 3:**
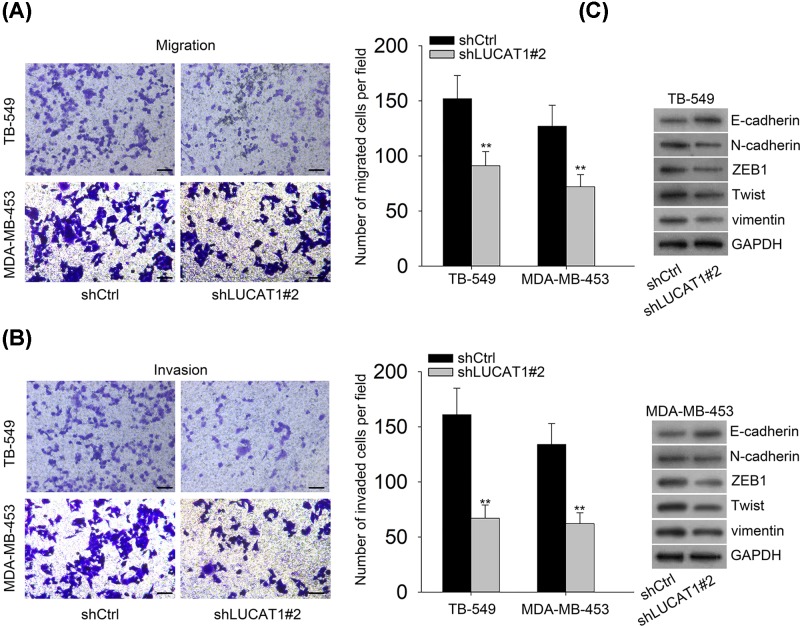
LUCAT1 suppression inhibits cell migration, invasion, and EMT of TNBC (**A,B**) Transwell assays were employed to evaluate cell migratory and invasive capacity. Scale bar = 100 μm. (**C**) Western blot analysis was conducted to detect the expression levels of E-cadherin, N-cadherin, ZEB1, Twist, and vimentin. All data were expressed as the mean ± SD, **P<0.01.

In order to further investigate whether LUCAT1 had impacts on cell migration and invasion, we performed the transwell assays and uncovered that inhibition of LUCAT1 contributed to a conspicuous reduce of cell migration and invasion in TNBC (Figure 3A,B). It is evident from emerging research that EMT plays a pivotal role in cell migration and invasion of human cancer [20]. To validate whether LUCAT1 was implicated in EMT process of TNBC, the Western blot analysis was employed to assess the level of EMT-related proteins. Our findings identified that cells transfected with shLUCAT1 dramatically promoted the expression of the epithelial marker E-cadherin, whereas resulted in the decreased expression of classic mesenchymal markers N-cadherin, ZEB1, Twist, and vimentin (Figure 3C). In summary, our data reveal that LUCAT1 knockdown is a suppressing regulator of cell migration, invasion, and EMT in TNBC.

### LUCAT1 directly interacts with miR-5702 and negatively regulates the expression of miR-5702

**Figure 4:**
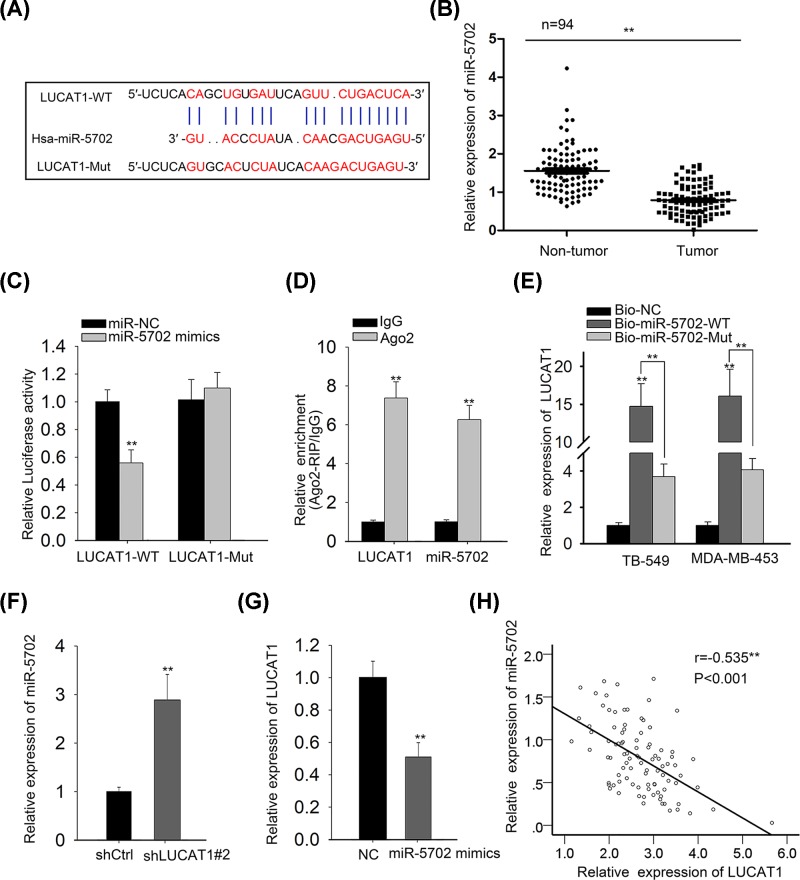
LUCAT1 directly interacts with miR-5702 and negatively regulates the expression of miR-5702 (**A**) The speculated binding sites of miR-5702 and LUCAT1 were predicted by LncBase Predicted v.2 database. (**B**) The miR-5702 expression in TNBC tissues and corresponding adjacent tissues were determined by qRT-PCR analysis. (**C–E**) The interaction between miR-5702 and LUCAT1 was estimated by luciferase reporter, RIP, and RNA pull-down assays. (**F,G**) qRT-PCR was utilized to verify the relationship between miR-5702 and LUCAT1. (**H**) Spearman’s correlation analysis was performed to analyze the correlation of miR-5702 and LUCAT1 expression in TNBC specimens. All data were expressed as the mean ± SD, **P<0.01.

Accumulating studies have illustrated that lncRNAs are deemed to serve as ceRNAs or molecular sponges for specific miRNAs to further modulate the biological role of them [21,22]. To explore the regulatory mechanism of LUCAT1 in TNBC, the bioinformatics analysis was utilized to conjecture the eligible miRNA which could directly bind to LUCAT1. Results delineated that miR-5702 contained binding sites of LUCAT1 (Figure 4A). Moreover, miR-5702 was reported to play a tumor suppressor in cancer and the expression of miR-5702 was predominantly decreased in TNBC samples compared with adjacent non-cancer samples (Figure 4B). In consideration of mentioned reasons, we selected miR-5702 as the further study object for following investigations. For sake of expounding the interaction of miR-5702 and LUCAT1, we conducted luciferase reporter assay, RIP assay, and RNA pull-down assay. As displayed in Figure 4C, the luciferase activity of LUCAT1-WT was only reduced by miR-5702, but there were no changes in that of LUCAT1-Mut and miR-NC. The RIP assay disclosed that LUCAT1 and miR-5702 were noticeably abundant in Ago2-containing beads in comparison to control group IgG (Figure 4D), indicating that miR-5702 could directly bind with LUCAT1. Likewise, RNA pull-down assay further confirmed that LUCAT1 was only pulled down by biotin-labeled miR-5702-WT (Figure 4E). Besides, our data from qRT-PCR assay suggested that LUCAT1 silence led to a significant rise of miR-5702 expression and ectopic expression of miR-5702 prominently restrained the expression level of LUCAT1 in TNBC cells (Figure 4F,G). Further correlation analysis was performed to elucidate the correlation of LUCAT1 and miR-5702 and results demonstrated that LUCAT1 was conversely associated with miR-5702 in TNBC tissues (Figure 4H). In other words, miR-5702 directly binds to LUCAT1 and is negatively modulated by LUCAT1.

### Repression of miR-5702 partially abrogates the inhibitory impacts of LUCAT1 knockdown on TNBC tumorigenesis

**Figure 5:**
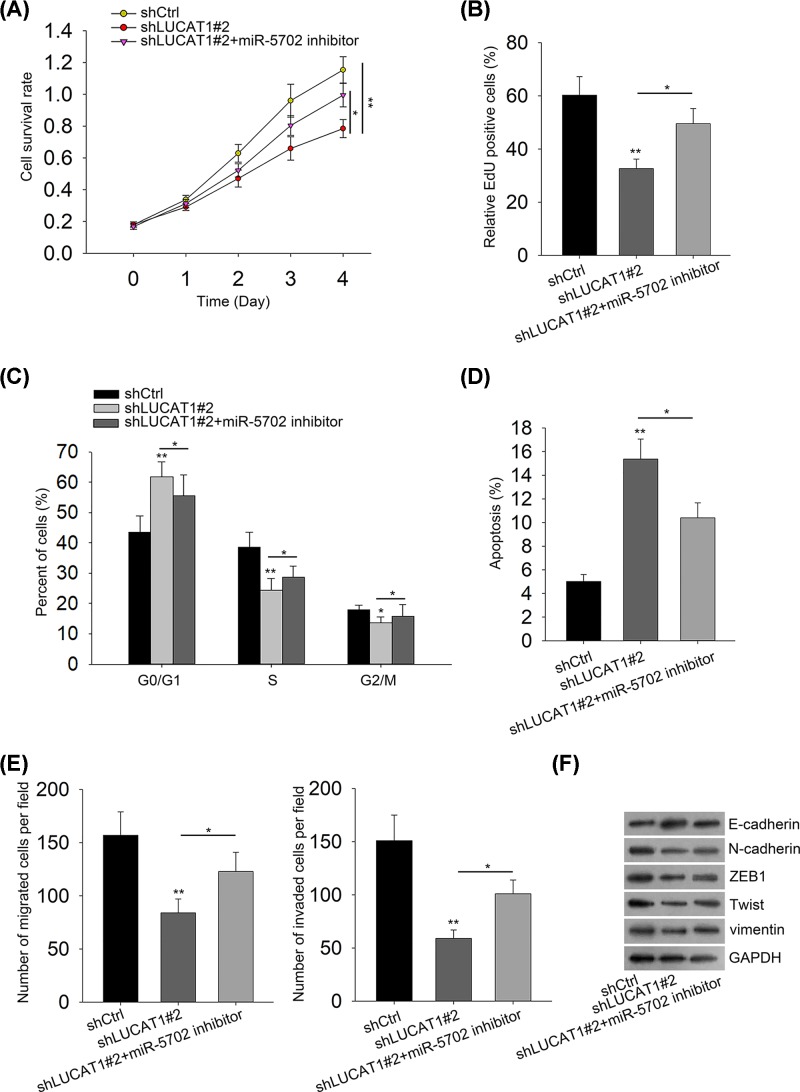
Repression of miR-5702 partially abrogates the inhibitory impacts of LUCAT1 knockdown on TNBC tumorigenesis (**A,B**) The CCK-8 and EdU assays were used to examine cell proliferation. (**C,D**) Cell cycle and apoptosis were analyzed by flow cytometry. (**E**) Cell migration and invasion were detected by transwell assays. (**F**) The expression of EMT-related proteins was assessed by Western blot analysis. All data were expressed as the mean ± SD, *P<0.05, **P<0.01.

To confirm whether LUCAT1 affected the progression of TNBC by regulating miR-5702 expression, rescue experiments were employed to validate the mechanism of LUCAT1 in TNBC. As demonstrated in Figure 5A,B, miR-5702 inhibitors promoted the decline of cell proliferation caused by depletion of LUCAT1. The cell cycle analysis exhibited that LUCAT1 silence induced cell cycle arrest at G0/G1 phase and then miR-5702 suppression enhanced the percentage of the G0/G1 phase, suggesting that miR-5702 knockdown retarded cell cycle arrest induced by silenced LUCAT1 and facilitated cell cycle progression (Figure 5C). Furthermore, we recognized that miR-5702 silence reversed the suppressing influences of LUCAT1 down-regulation on cell apoptosis (Figure 5D). Transwell assays showed that miR-5702 inhibitors counteracted the inhibition of cell migration and invasion induced by LUCAT1 depletion (Figure 5E). In the same way, knockdown of miR-5702 partially abolished the shLUCAT1-mediated regulation of EMT-relevant proteins (Figure 5F). Taken together, our results certify that miR-5702 inhibitors rescue the suppressing regulation of TNBC progression caused by LUCAT1 silence.

## Discussion

There is abundant evidence illustrating that lncRNA functions as a key regulator implicated in the mechanisms of tumorigenesis and has impacts on tumor formation, development, and metastasis in a variety of malignancies [23,24]. As the recent reports have shown, long non-coding RNA UPAT promotes cell proliferation via increasing UHRF1 expression in non-small cell lung cancer [25]. lncRNA CASC15 promotes melanoma progression by epigenetically regulating PDCD4 [26]. Overexpression of the long non-coding RNA CCAT1 promotes metastasis via epithelial-to-mesenchymal transition in lung adenocarcinoma [27]. Emerging studies have proven that LUCAT1 is an oncogene in human cancer. LUCAT1 promotes proliferation and invasion of clear cell renal cell carcinoma [28,29]. Additionally, LUCAT1 promotes tumorigenesis by controlling ubiquitination and stability of DNA methyltransferase 1 in esophageal squamous cell carcinoma [30]. However, the biological effect and molecular mechanism of LUCAT1 in TNBC progression need to be further verified, which may provide benefits for TNBC treatment.

In our study, we uncovered that the expression of LUCAT1 was dramatically up-regulated in TNBC samples and cell lines. Besides, our data revealed that high LUCAT1 expression was closely correlated with advanced stages and poor prognosis in TNBC. Further experiments were carried out to identify the role of LUCAT1 in TNBC and results certified LUCAT1 facilitated the progression of TNBC by promoting cell proliferation, cell cycle progression, and cell metastasis as well as inhibiting cell apoptosis. Increasing evidence is pointing that lncRNA competes for miRNA response elements (MREs) with the driver genes strongly relevant to cancer occurrence and development by acting as a ceRNA, which can impair the inhibitory impacts of miRNA on target genes, indirectly modulate the expression of target genes, and eventually involve in cancer regulation process [31]. We conjectured that LUCAT1 may serve as a ceRNA in TNBC. To further confirm this speculation, we utilized bioinformatics analysis to explore the potential genes possessing binding sites for LUCAT1. MiR-5702 was found to possibly bind with LUCAT1 by LncBase Predicted v.2 database and the expression of miR-5702 was remarkably reduced in TNBC tissues in comparison to non-cancer tissues. Furthermore, a recent study has implied that miR-5702 suppresses proliferation and invasion in non-small-cell lung cancer cells via posttranscriptional suppression of ZEB1 [32]. Based on the above reasons, miR-5702 was chosen to investigate the mechanism of LUCAT1 in the development of TNBC. Our findings elucidated that miR-5702 directly bound to LUCAT1 and was negatively regulated by LUCAT1. Besides, inhibition of miR-5702 partially rescued the suppressing influences of LUCAT1 silence on TNBC tumorigenesis.

In summary, our study provided the first evidence of the biological role and the potential molecular mechanism of LUCAT1 in TNBC progression. Our results proofed that LUCAT1 functioned as a cancer promotor by affecting cell proliferation, cycle, apoptosis, and metastasis of TNBC. Ultimately, we corroborated that LUCAT1 drove tumorigenesis and metastasis of TNBC via regulating miR-5702 expression. The LUCAT1/miR-5702 regulatory axis may provide innovative insights for development of therapeutic methods for TNBC. Logit-Lapnet method can help to identify biomarkers of TNBC [33], we will adopt this method to further validate our findings in the future.

## Abbreviations

CCK-8: Cell Counting Kit-8
EdU: ethynyldeoxyuridine
ER: estrogen receptor
HER2: human epidermal growth factor receptor-2
lncRNA: long non-coding RNA
PR: progesterone receptor
qRT-PCR: quantitative real-time PCR
RIP: RNA immunoprecipitation
TNBC: triple-negative breast cancer
